# Hearing dysfunction heralds an increase in non-motor burden and a worse quality of life in Parkinson’s disease: new insights from non-motor spectrum

**DOI:** 10.1007/s10072-024-07487-8

**Published:** 2024-04-01

**Authors:** Elena Garasto, Alessandro Stefani, Mariangela Pierantozzi, Matteo Conti, Arturo Moleti, Renata Sisto, Andrea Viziano, Claudio Liguori, Tommaso Schirinzi, Nicola Biagio Mercuri, Rocco Cerroni

**Affiliations:** 1 UOSD Parkinson’s Centre, Department of Systems Medicine, University of Rome “Tor Vergata”, Viale Oxford 81, 00133 Rome, Italy; 2 Department of Physics, University of Rome “Tor Vergata”, Via Della Ricerca Scientifica 1, 00133 Rome, Italy; 3 INAIL Research, Department of Occupational and Environmental Medicine, Epidemiology and Hygiene, Via Di Fontana Candida, INAIL Research, Via Di Fontana Candida, 1, 00078 Monteporzio Catone, Rome, Italy; 4 Department of Clinical Sciences and Translational Medicine, University of Rome “Tor Vergata”, Via Montpellier 1, 00133 Rome, Italy; 5 Department of Systems Medicine, University of Rome Tor Vergata, Rome, Italy; 6 IRCCS Fondazione Santa Lucia, Rome, Italy

**Keywords:** Auditory dysfunction, Hearing loss, Non-motor symptoms, Parkinson’s disease, Global burden of disease

## Abstract

**Background:**

Sensorial non-motor symptoms (NMSs) in Parkinson’s disease (PD) still lack appropriate investigation in clinical practice.

This study aimed to assess if and to what extent auditory dysfunction is associated with other NMSs in PD and its impact on patient’s quality of life (QoL).

**Methods:**

We selected patients with idiopathic PD, without other concomitant neurological diseases, dementia, or diagnosis of any audiological/vestibular disease. Demographic and clinical data were collected. Patients underwent otoscopic examination, audiological testing with pure tone audiometry (PTA) and distortion product otoacoustic emissions (DPOAEs) and completed Non-Motor Symptoms Scale (NMSS) and Parkinson’s Disease Questionnaires-39 (PDQ-39). ANCOVA and partial correlation analysis have been used for statistical analysis.

**Results:**

60 patients were enrolled and completed PTA and DPOAEs. 32 patients with hearing impairment (HI), assessed by PTA, (hearing threshold ≥ 25 dB) showed similar disease duration, motor impairment, and staging, compared to patients without HI, but higher scores both in NMSS and in PDQ-39, except for cardiovascular (CV), gastrointestinal (GI), urogenital (U) and sexual function (SF) of NMSS. In addition, DPOAEs showed a significant correlation with higher scores both in NMSS and PDQ-39, except for CV, SF, GI, U and perceptual problem subdomains of NMSS.

**Conclusion:**

This study demonstrated that PD patients with HI have a greater burden of NMS and lower related QoL and functioning. Our results highlight the importance to reconsider HI as a NMS, in parallel with the others. HI evaluation, even in asymptomatic patients, may reveal a wider pathology with a worse QoL.

## Introduction

Parkinson's disease (PD) is the second most common neurodegenerative disease, affecting more than 5 million people worldwide, with a further marked tendency to grow. Indeed, PD showed the highest increase in prevalence among neurological disorders between 1990 and 2015, and it’s estimated to double over the next 20 years [1]. For this reason, the need to stratify the pathology, identifying any subtypes and prognostic factors of the disease, is more and more pressing.

Nowadays, PD is considered a neurodegenerative multi-systemic syndrome, characterized by progressive motor impairment and a large spectrum of non-motor symptoms (NMSs).

Motor signs are commonly known to be bradykinesia, rest tremor, postural disturbances, and rigidity. Non motor features include a wide range of symptoms such as hyposmia, sensory symptoms, gastrointestinal and autonomic dysfunction, psychiatric features (e.g., depression, anxiety, psychosis), pain and fatigue, sleep disorders, and mild-to-severe cognitive impairment, many of which disabling and with a great impact on patient’s quality of life [2, 3].

Some NMSs like Rem behaviour disorders (RBD), constipation, hyposmia and depression may appear more than a decade before the onset of motor symptoms [4], characterizing the prodromal phase of the disease. Other NMSs develop and worsen as the disease progresses [5]; they also can sometimes fluctuate in the same way as motor symptoms, becoming more severe in “off state”[3]. In the last decades, research on NMSs has grown because of their potential role in early diagnosis and in understanding the pathogenesis of PD, as well as affecting the overall burden and the prognosis of the disease. The Lewy bodies (LB), pathological hallmarks of PD, have been found in other regions different from substantia nigra, such as myenteric plexuses, brainstem, and cerebral cortex [6, 7]. According to Braak’s hypothesis [8], their presence, distribution and progression are related to the appearance and severity of NMSs in the natural history of the disease. Moreover, not all NMSs underlie a dopaminergic dysfunction, but other neurotransmission pathways may be involved.

Among NMSs, the sensorial ones, which include pain, olfactory disturbances, and visual and auditory dysfunctions are often underestimated and untreated in clinical practice, although they may negatively impact patient quality of life [9]. Aetiopathogenesis and the features of these symptoms have not yet been fully clarified. Focusing on the auditory system, literature supported evidence of hearing impairment in PD [10, 11], although this finding has rarely been correlated with non-motor symptoms [12] and their association with clinical features still needs to be well defined [11, 13].

Hearing loss has been investigated through various means of testing, involving both peripheral pathways, that is, at the cochlear level [13–15], and central processing [12, 16, 17].

In our previous research, we focused on auditory dysfunction in PD [14, 15], showing worse hearing function in PD patients than controls; indeed, we found differences in multiple frequency bands of pure tone audiometry (PTA).

In this scenario, the present study aimed to investigate the possible correlation between auditory dysfunction – as shown of using PTA and distortion product otoacoustic emissions (DPOAE)—and the presence of other NMSs in a large cohort of PD patients.

Indeed, our purpose is to know if and to what extent auditory dysfunction is associated with a wider non-motor involvement in PD, with implications for the overall burden of disease and patients’ quality of life (QoL).

## Methods

### Patients selection and clinical evaluation

Patients were recruited during scheduled visits, from outpatients with PD afferent at the Movement Disorders Center of the Neurological Clinic of the University of Rome “Tor Vergata” between March 2019 and June 2021. All patients met the Movement Disorders Criteria for PD [18]; PD diagnosis was confirmed by at least two board-certified neurologists.

We excluded patients with other concomitant neurological diseases, psychiatric features, or any form of dementia (MMSE < 25). In line with the study design, patients are also required to have no history of any hearing loss (hereditary or acquired), previous head trauma or any diagnosis of audiological/vestibular disease. Patients with chronic exposure to noise, previous or current use of ototoxic drugs, presence of otosclerosis, tympanic perforation or severe comorbidities that may have interfered with the hearing function (hypertensive crisis, severe diabetes, peripheral vascular disease) were excluded. Patients younger than 50 years or older than 79 years were excluded, to limit the effect of age on auditory function.

Patients who met inclusion criteria underwent a preliminary neurological evaluation, followed by audiological anamnesis and otoscopic examination: motor impairment was assessed by Movement Disorder Society-Unified Parkinson’s Disease Rating Scale (MDS-UPDRS) Part III and disease progression by Hoehn and Yahr (H&Y) staging. All patients maintained their regular therapy and we tested them during “on state”. Levodopa equivalent daily dose (LEDD) was collected for each patient.

At baseline, the NMSs spectrum was assessed, for all patients selected, by the NMSs scale [19], while the quality-of-life aspects were scored by Parkinson’s Disease Questionnaires-39 (PDQ-39) [20]. In the NMSs scale, non-motor manifestations are divided into nine domains: cardiovascular, sleep/fatigue, mood/cognition, perceptual problems/hallucinations, attention/memory, gastrointestinal, genitourinary and miscellany symptoms.

PDQ-39 includes questions concerning mobility, activities of daily living, emotional well-being, stigma, social support, cognition, communication, and bodily discomfort; each one is represented in one of the eight different dimensions.

### Audiological testing

After the clinical evaluation including audiological history and otoscopy, patients underwent PTA, with stimulation frequencies ranging from 125 to 8000 Hz. For each patient, we took into consideration the mean hearing thresholds of both ears considering frequencies from 500 to 4000 Hz, as such frequency range is the most associated with human speech perception and according to the American Academy of Otolaryngology and American Council of Otolaryngology (AAO-ACO) guidelines, which define hearing loss as a mean hearing threshold of 25 dB or higher at the aforementioned frequencies.

Patients were then tested of using DPOAEs detection. DPOAEs are low-level sounds generated in the organ of Corti, in response to a selective auditory stimulation with two frequencies, f1 and f2. The main tones produced by intermodulation nonlinear distortion as a result of that stimulation are called distortion products and can be detected in the outer ear canal with a microphone. The main distortion product is recorded at frequency 2f1-f2, although it is clinically associated with inner ear function at frequency f2.

DPOAEs mainly refer to the state of functionality of the outer hair cells, with great sensitivity to early detect sensorineural hearing loss [21]. DPOAE spectra were recorded with high frequency-resolution and filtered to unmix the distortion and reflection components [22] and to remove a large fraction of random noise. To guarantee that noise contribution to the estimated DPOAE component level was not significant, we included in the study only data for which either the noise was below a given threshold, or the signal-to-noise ratio (SNR) was higher than 3 dB. We considered as outcome variables the level of the unmixed distortion component, averaged in third-octave bands, and the average audiometric hearing level in the range from 500 to 4000 Hz.

### Study design and ethical committee

We performed a cross-sectional study, selecting from our cohort PD patients with and without hearing impairment, evaluated through PTA and DPOAEs. Then, we correlated the hearing impairment with NMSs and QoL, as a sign of a more severe and widespread disease. All patients received an extensive disclosure of the study’s purposes and risks, and, therefore, gave written informed consent. The study was approved by the local ethics committee (protocol no. 7/18, February 7th, 2018).

### Statistical analysis

All statistical analyses were performed using the statistical software SPSS (version 25, IBM, USA). For comparison between groups, the Student t-test was used for normally distributed continuous data, and the χ^2^ test for the other group differences. Analysis of covariance (ANCOVA) was used to compare scores of the two examined scales among patients with and without hearing impairment, assessed by PTA, where each score was considered as the dependent variable, the presence of hearing impairment as a fixed factor, and age and gender as covariates.

Instead, DPOAE measurements were considered as continuous variables and partial correlation analysis was used to assess the correlation between each subdomain’s score of the two scales and DPOAEs, with age and gender as control variables. A significance criterion of *p* < 0.01 was adopted.

## Results

### Demographics

Sixty-five patients, taking into account inclusion/exclusion criteria, were enrolled. After audiological anamnesis and examination, five of them were excluded: three reported previous use of ototoxic drugs and two reported professional noise exposure. Thus, 60 patients were enrolled (M = 53.3%%, F = 46.7% mean age of 65.4 ± 7.4).

### Audiometric data results

Concerning PTA, subjects were considered as affected by hearing loss if the mean hearing threshold was ≥ 25 dB, according to the AAO-ACO guidelines. Thirty-two patients showed the presence of hearing impairment (PD patients with hearing impairment, referred to as PDwHI), and 28 showed normal hearing function (PD patients without hearing impairment, referred to as PDwoHI). As shown in Table 1, these two groups were similar for age, gender and clinical variables, such as motor impairment (MDS-UPDRS III), motor phenotypes, staging (H&Y), disease duration and therapy (LEDD). Notably, analysis of covariance, adjusted for age and gender, showed that PDwHI have higher scores of NMSS and PDQ-39 than PDwoHI. A statistically significant difference was found in almost all subdomains, except for cardiovascular, urogenital, gastrointestinal and sexual function domains of the NMSs scale. Results with a mean and standard deviation of single subdomains and corresponding p-value are shown in Table 2.

**Table 1:** Clinical and demographic characteristics of patients with and without hearing impairment, defined by PTA

	PDwHI PTA ≥ 25 dB *n* = 32	PDwoHI PTA < 25 dB *n* = 28	Sig (*p* value)
Age (years)	66.9 ± 6.8	63.7 ± 7.7	0.095
Gender	M: 66%; F:34%	M: 40%; F60:%	0.041
M = male; F = female			
Disease duration (months)	63.7 ± 47	66.9 ± 50.7	0.804
MDS-UPDRS III	25.47 ± 9.6	21.5 ± 9.5	0.069
H&Y on	2.09 ± 0.8	2.36 ± 0.9	0.245
LEDD	453 ± 350	501 ± 342	0.595
Motor subtypes (n.)			
Tremor-dominant	8	4	
Akinetic-rigid	14	16	0.490
Mixed	10	8	

Results are reported as mean ±  standard deviation or as counts (%)

*PDwHI* PD patients with hearing impairment, *PDwoHI* PD patients without hearing impairment, *PTA* pure tone audiometry, *H&Y* Hoehn;Yahr, *UPDRS* Unified Parkinson’s Disease Rating Scale, *LEDD* levodopa equivalent daily dose

*Statistically significant data (*p* < 0.01)

**Table 2:** Comparison of NMSS and PDQ-39 subdomains in patients with and without hearing impairment, defined by PTA

	PDwHI n.32	PDwoHI n.28	Sig
PDQ-39 mobility	48.9 ± 15.3	29.0 ± 18.2	0.000*
PDQ-39 ADL	49 ± 18.8	27.2 ± 21.2	0.000*
PDQ-39 emotional well-being	40.8 ± 13.5	27.2 ± 13.2	0.000*
PDQ-39 stigma	37 ± 14.4	15.1 ± 15	0.000*
PDQ-39 social support	27.1 ± 10.6	11.6 ± 11.4	0.000*
PDQ-39 cognition	39.3 ± 15.5	27.7 ± 13.6	0.005*
PDQ-39 communication	40.6 ± 11.9	20.6 ± 15.6	0.000*
PDQ-39 bodily disconfort	40.5 ± 14.2	21.2 ± 13.3	0.000*
PDQ-39 total	40.3 ± 7.2	22.7 ± 5.9	0.000*
NMS cardiovascular	5.7 ± 5.1	4.7 ± 5.3	0.48
NMS sleep/fatigue	15.9 ± 3.4	10.1 ± 2.7	0.000*
NMS mood/cognition	17.1 ± 8.7	9.4 ± 7.7	0.000*
NMS perceptualproblem/hallucinations	4.9 ± 5.8	0.9 ± 1.4	0.002*
NMS attention/memory	10.2 ± 5.1	6,5 ± 3.9	0.006*
NMS gastrointestinal	8.3 ± 4.6	5.8 ± 2.7	0.054
NMS urogenital	11.7 ± 8.3	6.6 ± 5.9	0.029
NMS sexual function	6.6 ± 3.8	5.1 ± 4.4	0.195
NMS miscellaneous	10.7 ± 4	4 ± 1.4	0.000*
NMS total	91.6 ± 15.8	52.1 ± 10.8	0.000*

*PDwHI* PD patients with hearing impairment, *PDwoHI* PD patients without hearing impairment, *PDQ* Parkinson’s Disease Questionnaires, *NMS* non-motor symptoms

Data are reported as average values and standard deviations. Results are adjusted for age and gender

*Statistically significant data (*p*<0.01)

### DPOAEs data results

We considered DPOAEs outcome as a continuous variable since there is no normative threshold level defined in current literature to determine hearing impairment. Anyway, it must be considered that higher DPOAEs correspond to better hearing function. To give more consistency to our results we performed a Pearson correlation analysis showing indirect correlation between DPOAE variables and hearing threshold values by PTA (r = -0.8, *p* = 0.000). The DPOAE noise rejection criterion reduced the number of applicable DPOAEs to 56 subjects.

When correlation with scale outcome was investigated, adjustment for age and gender was considered. We found significant inverse correlations between DPOAE and PDQ-39 mobility (*p* = 0.001), PDQ-39 ADL (*p* = 0.001), PDQ-39 emotional well-being (*p* = 0.000), PDQ-39 stigma (*p* = 0.000), PDQ-39 social support (*p* = 0.000), PDQ-39 cognition (*p* = 0.002), PDQ-39 communication (*p* = 0.000), PDQ-39 bodily discomfort (*p* = 0.000), PDQ-39 total (*p* = 0.000), NMS sleep/fatigue (*p* = 0.000), NMS mood/cognition (*p* = 0.009), NMS attention/memory (*p* = 0.000), NMS miscellaneous (*p* = 0.000), NMS-total (*p* = 0.000). No significant correlation was found with cardiovascular, gastrointestinal, perceptual problems, sexual function and urogenital subdomains of the NMSs scale, although there was a tendency for NMS perceptual problems. Results are shown in Table 3.

**Table 3:** Correlation between DPOAEs and subdomain scores of NMSs scale and PDQ-39

	DPOAEs	
	Coeff. R	Sig
PDQ-39 mobility	-0.44	0.001*
PDQ-39 ADL	-0.453	0.001*
PDQ-39 emotionalwell-being	-0.5843	0.000*
PDQ-39 stigma	-0.509	0.000*
PDQ-39 social support	-0.47	0.000*
PDQ-39 cognition	-0.422	0.002*
PDQ-39 communication	-0.532	0.000*
PDQ-39 bodilydisconfort	-0.67	0.000*
PDQ-39 total	-0.698	0.000*
NMS cardiovascular	0.086	0.538
NMS sleep/fatigue	-0.540	0.000*
NMS mood/cognition	-0.358	0.009*
NMS perceptualproblem/hallucinations	-0.279	0.043
NMS attention/memory	-0.478	0.000*
NMS gastrointestinal	-0.181	0.194
NMS urogenital	-0.168	0.230
NMS sexual function	-0.14	0.316
NMS miscellaneous	-0.529	0.000*
NMS total	-0.611	0.000*

The coefficient and *p*-value of partial correlation analysis are shown. Results are adjusted for age and gender

*DPOAE* distortion product otoacoustic emissions, *PDQ* Parkinson’s Disease Questionnaires, *NMS* non-motor symptom

*Statistically significant data (*p*<0.01)

**Fig. 1:**
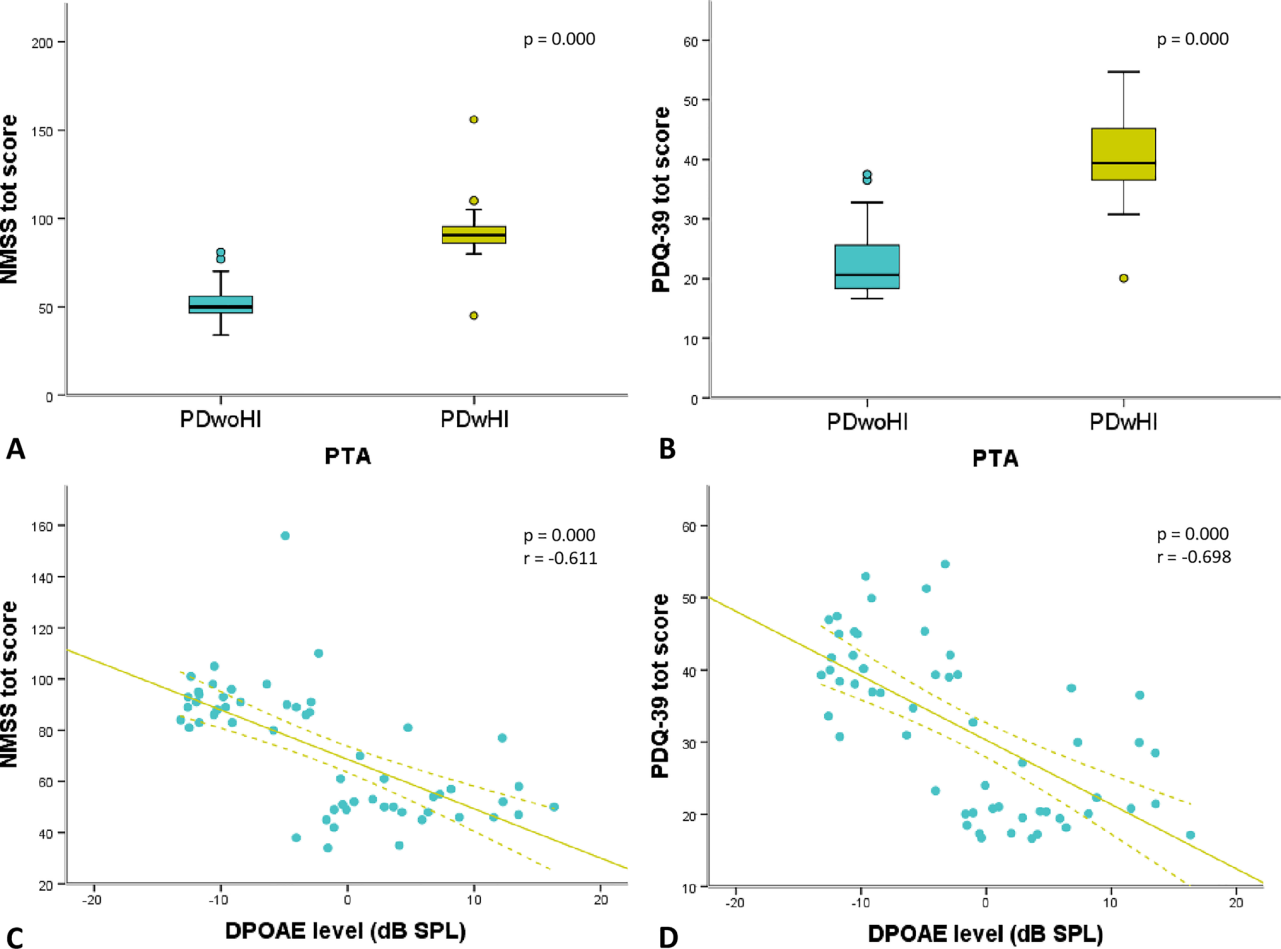
Graphic representation of the main results: on the top, differences of NMSS total score (**A**) and PDQ-39 total score (**B**) between PDwoHI and PDwHI are shown, when assessed by PTA; the lower row represents DPOAE correlation with NMSS (**C**) and PDQ-39 total score (**D**)

Our main results, concerning both PTA and DPOAEs, are illustrated in Fig. 1.

## Discussion

The present study shows that PD patients with worse hearing function have greater involvement of non-motor symptoms and lower related quality of life, than PD patients with better hearing function, regardless of disease staging and motor impairment. Also, these findings appear strongly significant.

The presence of auditory dysfunction in PD seems to underlie a greater disease burden, with a more widespread non-motor involvement that inevitably adversely affects the lives of patients.

According to our findings, hearing dysfunction in PD seems to be strongly related to many NMS domains, especially sleep/fatigue, mood/cognition, attention/memory, and miscellaneous symptoms (which include general pain, swelling, weight loss and hyposmia), as well as to total score of NMSs scale. Taking into account several studies, included our previous work, documented PD patients as having worse hearing dysfunction compared to controls [10–12], here we added a novelty element in terms of association of HI with other well-known NMSs. Furthermore, results are confirmed by both hearing assessment tools (PTA and DPOAEs) which accurately evaluate hearing function, but in different ways.

Several non-motor symptoms have been related to each other in literature. Among them, fatigue, considered halfway between motor and non-motor symptoms, have been strictly correlated to NMSs, in particular with mood (apathy, anxiety, anhedonia), sleep disorders (excessive daytime sleepiness and sleep difficulties), and cardiovascular autonomic dysfunction [23]. Mao et al. [24], among sensory comorbidity, explored pain in PD, comparing motor and non-motor symptoms in PD patients with and without pain, demonstrating that patients with pain suffer from more frequent and extended non-motor symptoms, evaluated through NMS questionnaires, such as weight loss, memory disturbances, depression, insomnia, rapid eye movement disorder, and swelling.

These works, like many others in literature, stress the concept of PD as a multi-systemic disease, with the involvement of multiple cortical-subcortical areas other than basal ganglia, multiple neurotransmission pathways other than dopaminergic one [25], and multiple systems other than the nervous one. For example, the presence of Lewy bodies and Lewy neurites, pathological hallmarks of PD, has been found in the brainstem as well as in the intestinal wall [6] and in the skin [26]. Moreover, several nuclei located in the brainstem can be affected: among them, the dorsal motor vagus nucleus, which acts as the main parasympathetic nucleus controlling the enteric system and gastrointestinal motility, is involved in PD pathology from the very early stage [8]. Degeneration of the dorsal raphe, coeruleus, and pedunculopontine nuclei, affecting serotoninergic, noradrenergic, and cholinergic neurotransmission, have also been described in PD pathogenesis and associated with the presence of pain, mood disorder, fatigue and sleep disturbances, including excessive daytime sleepiness and rem-behavior disorders [27]. Finally, nigro-striatal degeneration itself and dopamine dysregulation have been linked to the genesis of neuropsychiatric symptoms, sleep disturbances, and sensory and autonomic symptoms [28].

In line with these findings, according to our research, the pathogenesis of auditory dysfunction in PD seems to be multifactorial; indeed, it is conceivable that structures such as the cholinergic medial olivocochlear bundle, and descending pathways from the pedunculopontine tegmental nucleus, other than dopaminergic synapses which reside mainly in the lateral olivocochlear bundle, may underlie hearing loss.

In this study, we did not find a significant correlation between hearing disturbances and some NMSs, mainly concerning autonomic dysfunction (sexual function, gastrointestinal, urinary and cardiovascular domains). This is in our opinion an interesting finding, which could lead to better subtyping the disease, based on differential non-motor involvement. We speculated on the idea of two different PD-related phenotypes based on NMSs involved: the first one, mainly characterized by neuropsychiatric and cognitive dysfunctions, involving sensory disturbances and sleep imbalance (“central” non-motor phenotype), and a second one, with predominant autonomic features, with a greater involvement of the sympathetic and parasympathetic systems than the central pathways (“neurovegetative” non-motor phenotype). This idea requires to be confirmed by further and targeted studies. However, it seems to be in accordance to recent scientific topic that underlie the precence of two different physiopatological precesses in PD (brain first vs body first) [29].

In addition, we investigated the quality of life and functioning of patients, through the PDQ-39 questionnaires, which showed higher scores when hearing impairment was assessed. This reinforces the idea that hearing dysfunction could seriously affect the patient’s quality of life, already compromised in PD.

We found no significant differences between patients with and without hearing dysfunction (by both DPOAEs and PTA outcome), concerning disease duration, motor impairment, motor subtypes, LEDD and staging of disease, confirming findings from a previous study by our group [15], but in contrast with other works: Vitale et al., i.e., found a significant correlation between PTA and H&Y [10], furthermore Pisani et al. correlated DPOAE dysfunction with clinical severity and PTA with disease duration, although in an exiguous number of patients [13]. However, most of our patients were on H&Y stage 1–2, and their evaluation in “best on” could have interfered with these findings; nevertheless, in our opinion, this is not a limitation of the study, since the similarity of these motor variables between the two groups allowed us to compare non-motor symptoms without disease-related confounding, which was the main objective of our study. Moreover, while in our recent study we focused on the relationship between cochlear dysfunction, lateralization of motor impairment, and striatal DAT availability [30], in this study we switched our focus to the relationship between auditory dysfunction and other NMSs, with implications in QoL.

Being a cross-sectional study, in this work we were not able to assess the onset and progression of hearing impairment and its temporal correlation with other non-motor symptoms. In future studies the prevalence of hearing loss in the prodromal phase of PD should be investigated; longitudinal studies could also evaluate the progression of inner ear involvement in PD and better clarify its association with disease progression and staging. A limitation of this study was the absence of a control group of healthy subjects, although it was not deemed imperative since we aimed to see the possible association of hearing dysfunction with other non-motor symptoms and quality of life in PD patients.

On the contrary, a point of strength was that the neurologist who performed questionnaires was blinded to the result of audiological performances. Furthermore, DPOAE evaluations gave us an objective tool to assess hearing function, regardless of patient collaboration.

Finally, we confirmed the idea that hearing impairment is part of the complex features of this neurodegenerative disease, in agreement with previous literature showing a higher prevalence of hearing loss in PD compared to controls [10, 14, 15]. Moreover, the evaluation of hearing function in PD must not be neglected, and hearing loss should be investigated as a non-motor symptom, although auditory dysfunction can be initially asymptomatic [12].

It should be noted that using the clinical dichotomous definition of hearing impairment, which is based on a rather high hearing level threshold, may overlook the early symptoms of hearing impairment, particularly in relatively young subjects. The use of a continuous outcome variable as the DPOAE level, particularly sensitive to mild levels of damage to the cochlear amplifier, could be particularly useful for the early detection of these symptoms.

In conclusion, our data support the presence of extensive comorbidity between the NMSs here evaluated, and hearing dysfunction, except for the dysautonomic symptoms. This can be explained by the combination of a multifocal alpha-synuclein pathology and a direct influence of hearing function on some neuro-psychological aspect as cognition, mood, social stigma, and communication. Our study points to considering hearing impairment in PD as a non-motor symptom, in parallel with the others and highlights the importance of its evaluation, even in asymptomatic patients, since it can be a determinant of the quality of patients’ lives.

## Data Availability

Anonymized data can be requested upon reasonable request to the corresponding author.
